# Antiphotoaging Potential of Extracts of Yin-Tonic Herbal Medicine in Skin Cell and Human Skin Equivalent

**DOI:** 10.1155/2020/8881270

**Published:** 2020-11-20

**Authors:** Yun-mi Kang, Min-gyu Seo, Kyou-young Lee, Hyo-jin An

**Affiliations:** ^1^Department of Pharmacology, College of Korean Medicine, Sangji University, Wonju, Republic of Korea; ^2^Department of Korean Ophthalmology and Otolaryngology and Dermatology College of Korean Medicine, Sangji University, Wonju 26339, Gangwon-do, Republic of Korea

## Abstract

Yin-tonic herbal medicines have been shown to possess properties that make skin healthy by nourishing within various organs of the body. However, the antiphotoaging effect of these medicines on the skin has not been fully studied. Photoaging occurs with prolonged sun exposure and causes skin damage and aging, with depletion of the dermal extracellular matrix and chronic alterations in skin structure, such as wrinkles. In this study, we assessed the antiphotoaging effects of eight yin-tonic herbal medicines on human skin cells and skin equivalents. The levels of type I procollagen and matrix metalloproteinase-1 (MMP-1) in ultraviolet B- (UVB-) irradiated CCD-986sk fibroblasts were measured, and then three medicines were chosen based on screening results. Using UVB-irradiated human skin equivalents, we evaluated the effect of three yin-tonic herbal medicines on histological changes of skin, epidermal and dermal thickness, and MMP-1 production. Furthermore, we observed collagen fiber content and protein expression of filaggrin in UVB-irradiated human skin equivalents. Yin-tonic herbal medicines increased type I procollagen levels and decreased the production of MMP-1 in UVB-irradiated CCD-986sk fibroblasts. The three selected yin-tonic herbal medicines recovered the collagen content and filaggrin expression via MMP-1 downregulation in UVB-irradiated human skin equivalents. Our results show that yin-tonic herbal medicines can prevent skin photoaging by reduction of MMP-1 levels and increasing the expression of moisturizing factors. Based on these results, we suggest that yin-tonic herbal medicines have the potential to be used as helpful agent for skin photoaging.

## 1. Introduction

Skin aging is a complex biological process that progresses as a person ages or is exposed to sun. Skin aging can be divided into two types, intrinsic or chronological aging and extrinsic or photoaging, based on the physiological and environmental factors, respectively [1]. The intrinsic aging is influenced by internal factors including gene expression, decline of hormones, and skin disorders involving cutaneous barrier dysfunctions. The major external factors are ultraviolet radiation (UVR), smoking, air pollution, and toxins [2]. In particular, UVR is the primary factor responsible for skin damage and characterizes a process known as photoaging. UVR is defined as that portion of the electromagnetic spectrum between X-rays and visible light. Although UV energy has beneficial effect on the skin, UV absorption by the skin involved in skin pathology such as aging cancer, and autoimmune responses. According to wavelength, which ranges from 100 to 400 nm, UVR is categorized into three primary types: ultraviolet A (UVA), ultraviolet B (UVB), and ultraviolet C (UVC) [3, 4].

UVA (320–400 nm) is considered to have a minor effect on skin, although studies have shown that these rays can penetrate deep into the skin. UVC radiation (100–290 nm) is almost completely absorbed by the ozone layer and does not affect the skin. UVB (290–320 nm) has high energy to cross the epidermis and reach the upper dermis where it interacts with cellular chromophores, leading to increased oxidative stress and DNA damage [5]. In other words, both UVA and UVB cause skin damage, but UVB can directly interact with DNA of the cell and induces base structural DNA damage, resulting in negative effects such as pigmentation, inflammation, cell apoptosis, burns, photoaging, and skin cancer. Thus, it is considered the most harmful rays of sunlight to skin [4, 6]. Morphologically, photoaged skin shows a variety of clinical characteristics, including fine and coarse wrinkles, sallow discoloration, pigmentation changes, telangiectasia, increased fragility, dryness, and rough skin texture [7]. Photoaging of the skin is a complex biological process affecting various layers of the skin with major damage seen in the connective tissue of the dermis. Histological studies have revealed major alterations in dermal connective tissue, characterized by disorganized and damaged collagen fibrils, which make up the bulk of dense skin connective tissue [8].

In the extracellular matrix (ECM), type I collagen is the predominant form of structural fibrous proteins. Procollagen, collagen precursor molecules, are synthesized by dermal fibroblasts and secreted into extracellular spaces, where it is enzymatically processed to mature collagen. Mature collagen pack together to form fibrils, which are largely responsible for the tensile strength and mechanical resilience of connective tissues in skin. The loss of collagen in the ECM can be attributed to the reduced synthesis and abnormal breakdown of dermal collagen [9]. Under the exposure of UV radiation, the upregulation of matrix metalloproteinases (MMPs) enzymes secreted by keratinocytes, fibroblasts, and other cells, promotes the breakdown of collagen by increasing activator protein-1 and decreasing collagen synthesis [10]. MMPs are a family of zinc-containing endopeptidases that are responsible for the degradation of ECM proteins. To date, the MMP family consists of 25 members, 24 of which are expressed in mammals. MMPs are divided into collagenases, gelatinases, stromelysins, and membrane-type MMPs [8]. UV light is known to induce the expression of MMP-1, MMP-2, and MMP-9 and MMP inhibition may be a promising strategy to prevent photoaging [11]. The dermal collagen fibrils in the ECM are hydrolyzed by MMPs in a process initiated by the collagenase MMP-1, a major collagenolytic enzyme [12].

The concept of yin and yang is a fundamental principle of traditional medicine and the harmony of body can be understood through the philosophy of yin and yang [13]. According to the relationship between yin and yang, health is achieved when the two are in balance, while disharmony develops and disease occurs when yin or yang is out of balance. Yin-yang attribution is also used to define the nature of herbal medicines, as well as diagnosis from the description of patient symptoms. Less yin in skin is believed to lead to symptoms such as skin dryness; thus, skin texture and elasticity can be improved by nourishing yin, replenishing blood, and activating the flow of fluids. There are specific herbal medicines that exert yin and yang tonic function [14]. Because those medicines possess tonifying, nourishing, supplementing, and strengthening abilities, they are used to treat deficiency syndromes [15]. According to traditional medical literature, including the Donguibogam, eight types of yin-tonic herbal medicines which have nourishing, nutritious, and moistening properties were selected. The present study was designed to determine the potential antiphotoaging effect of yin-tonic herbal medicines. Herbal extracts are primarily added to cosmetic formulations due to their beneficial antioxidant and anti-inflammatory properties. In this study, we proposed the antiphotoaging effect of yin-tonic herbal medicines using an *in vitro* and human skin equivalent *in vivo* model.

## 2. Materials and Methods

### 2.1. Chemicals and Reagents

Iscove's modified Dulbecco's medium (IMDM), Dulbecco's Modified Eagles medium (DMEM), Dulbecco's phosphate-buffered saline (DPBS), fetal bovine serum (FBS), penicillin, and streptomycin were purchased from Life Technologies Inc. (Grand Island, NY, USA). 3-(4,5-dimethylthiazol-2-yl)-2,5-diphenyltetrazolium bromide (MTT) and dimethyl sulfoxide (DMSO) were purchased from Junsei Chemical Co., Ltd. (Tokyo, Japan). Procollagen Type I C-peptide (PIP) EIA kit was purchased from Takara Bio Inc. (Kusatsu, Shiga, Japan). Human Total MMP-1 DuoSet ELISA kit was obtained from R&D Systems (Minneapolis, MN, USA).

### 2.2. Preparation of Samples

The eight yin-tonic herbal medicines used in this study are listed in Table 1. Dried plants were purchased from Nanumherb (Yeongcheon, Korea), Hanherb (Guri, Korea), ShinHung (Yeosu, Korea) and extracted with 30% ethanol and the extract was concentrated under reduced pressure. The decoction was filtered, lyophilized, and stored at 4°C. The yield of the dried extract from the starting crude materials isdescribed in Table 1. To prepare samples for the *in vitro* experiment, the extract powder that resulted from the drying process was dissolved in distilled water.

### 2.3. Cell Culture

CCD-986sk fibroblasts were purchased from Korea Cell Line Bank (KCLB, Seoul, Republic of Korea). The cells were incubated at 37°C in IMDM supplemented with 10% FBS, penicillin (100 U/mL) and streptomycin (100 *μ*g/mL) in a humidified atmosphere with 5% CO_2_. HaCaT keratinocytes were provided by Professor Jae-Young Um (Kyung Hee University, Republic of Korea) and were incubated at 37°C in DMEM supplemented with 10% FBS, penicillin (100 U/mL), and streptomycin (100 *μ*g/mL) in a humidified atmosphere with 5% CO_2_.

### 2.4. Cell Viability

Cell viability was assessed using the MTT assay. Briefly, cells were treated with each of the samples and incubated for 24 h, followed by incubation with MTT solution (5 mg/mL) for 4 h at 37°C. After discarding the supernatant, the insoluble formazan product was dissolved in DMSO. Cell viability was measured at 540 nm using a microplate reader (Titertek Multiskan, Huntsville, AL, USA).

### 2.5. UV Irradiation to CCD-986sk Fibroblasts

As a UVB source, a UVB lamp (BLX crosslinker, Vilber BIO-LINK, France), having an emission spectrum of 280–320 nm and the spectral peak of UVB, was set at 312 nm in this study. The programmed microprocessor controlled the constant UVB light emission with precise irradiation in energy or time. After CCD-986sk, fibroblasts were seeded and precultured with serum-free medium for 24 h and then treated with samples in serum-free medium for 24 h. Before UV irradiation, the cells were washed twice with DPBS and submerged in a minimum volume of DBPS, followed by exposure to UVB light using the UVB lamp. The UVB dose of 20 mJ/cm^2^ was used according to the previous reports [16–18]. After UV irradiation, the cells were washed with DPBS and cultured for 24 h in serum-free medium. Nonirradiated control cells were maintained under the same culture conditions without UVB exposure.

### 2.6. Type I Procollagen and MMP-1 Assay

Harvested cell supernatants and culture media from the human skin equivalent model were used for the experiments. Type I procollagen and MMP-1 production measurement was performed using an ELISA kit in accordance with the manufacturer's protocol.

### 2.7. Human Skin Equivalent Preparation and MTT Assay

Neoderm^®^-ED which is a human skin equivalent model was purchased from TEGO Science (Seoul, Korea). Briefly, human dermal fibroblasts were cultured in the collagen matrix for 1 day before keratinocytes were seeded on top of the collagen matrix and cocultured for 4 days. The keratinocytes were then lifted, and the human dermal fibroblasts block was exposed to the air. Samples were treated for 1 h, and the cells were irradiated with 50 mJ/cm^2^ solar UVB twice daily for 8 days. The skin equivalent was incubated at 37°C in an atmosphere of 5% CO_2_. The MTT assay was performed in accordance with the manufacturer's protocol. Briefly, human skin equivalent was pretreated with samples and incubated for 24 h, followed by incubation with MTT solution (0.3 mg/mL) for 3 h at 37°C. Separated skin equivalent samples were taken using 8 mm biopsy punch and were decolorized by stirring in 0.04 N HCl-isopropanol solution for 4 h. Cell viability was measured at 570 nm using a microplate reader.

### 2.8. UV Irradiation to Human Skin Equivalent

UVB irradiation and treatment with the samples were performed according to a method previously described [19–22] with some modifications. In short, the human skin equivalent was treated with yin-tonic herbal medicines for 1 h, and the skin equivalent was irradiated with 50 mJ/cm^2^ UVB twice daily for 8 days. The skin equivalent was incubated at 37 °C in an atmosphere of 5% CO_2_.

### 2.9. Histopathological and Immunohistochemical (IHC) Analysis

Human skin equivalent samples were fixed in 10% buffered formalin, embedded in paraffin, sectioned into 4 *μ*m thick, and hematoxylin and eosin (H&E) and Masson's trichrome staining were performed. For immunohistochemical staining, a portion of the skin samples from the back of mice in each group was fixed in 10% formalin. After paraffin embedding, sections were cut and the slides were deparaffinized by xylene, rehydrated in ethanol, and rehydrated in water. Endogenous peroxidase activity was blocked using 0.6% H_2_O_2_ in 50% methanol, and the slides were then treated with 0.3 triton in DPBS for permeabilization and problocked with 10% normal goat serum (NGS) for 1 h, followed by overnight incubation with a specific antibody at 4°C. The sections were then washed and incubated with horseradish peroxidase-conjugated secondary antibodies for 1 h at room temperature. The activity was visualized with 3,3′-diaminobenzidinechromogen and counterstained with H&E. Pathological changes of all stained skin sections were observed using a DM IL LED microscope (Leica, Wetzlar, Germany) and photographed using a DFC295 (Leica, Wetzlar, Germany). Digital images were taken from each slide (2 per group) and measured using Leica Application Suite (Leica, Wetzlar, Germany).

### 2.10. Statistical Analysis

Data are expressed as the mean ± SD for triplicate experiments. Statistically significant values were compared using ANOVA and Dunnett's post hoc test, and *p* values <0.05 were considered statistically significant. Statistical analysis was performed using SPSS statistical analysis software (version 19.0, IBM SPSS, Armonk, NY, USA).

## 3. Results and Discussion

### 3.1. Evaluation of Cytotoxic Effects of Eight Yin-Tonic Herbal Medicines on HaCaT Keratinocytes and CCD-986sk Fibroblasts

To test whether the eight yin-tonic herbal medicines could affect cell viability on skin cells, an MTT assay was used. The cell cytotoxicity of the eight yin-tonic herbal medicines against human keratinocytes (HaCaT cells) and human skin fibroblasts (CCD-986sk cells) is shown in Tables 2 and 3. Treatment with up to 500 *μ*g/mL doses of the eight yin-tonic herbal medicines did not affect the viability of both of the cell lines, rather skin constituting cells were proliferated by the treatment. Thus, the extracts concentrations 125, 250, and 500 *μ*g/mL were used for subsequent studies.

### 3.2. Effect of Eight Yin-Tonic Herbal Medicines on Procollagen and MMP-1 Production in UVB-Irradiated CCD-986sk Fibroblasts

**Figure 1:**
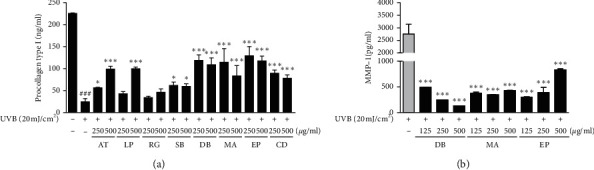
Effect on procollagen and MMP-1 production of eight yin-tonic herbal medicines on UVB-irradiated CCD-986sk fibroblast. (a) Type I procollagen and (b) MMP-1 production were measured by ELISA. CCD-986sk fibroblast was pretreated with samples for 24 h and then irradiated with UVB at a rate of 20 mJ/cm^2^. The cell supernatants were obtained for assay. ^###^*p* < 0.001 versus the control group; ^*∗*^*p* < 0.05 and ^*∗∗∗*^*p* < 0.001 versus UVB-irradiated group or nonirradiated group.

As a key indicator of photoaging, a decreased collagen production is observed in dermal fibroblasts. UV irradiation causes alternations of dermal collagen through stimulation of collagen breakdown, resulting in a fragmented and disorganized collagen fibers [23]. Therefore, the collagen biosynthesis degree within cells can be measured by the amount of the procollagen present. To determine the effects of the eight yin-tonic herbal medicines on UVB-damaged skin, we measured cellular levels of procollagen type I expression in CCD-986sk fibroblasts. UV-irradiated fibroblasts had lower procollagen type I expression than unexposed cells. We found that treatment with the eight yin-tonic herbal medicines increased procollagen type I production compared with UVB-irradiated fibroblasts alone (Figure 1(a)). Collagen, the major component of skin, is degraded by the enzyme collagenase. Inhibition of collagenase activity delays the process of forming precollagen fibers and subsequently the wrinkling process [24]. In addition, MMPs are overexpressed in senescent fibroblasts [25]. Among the eight yin-tonic herbal medicines, the treatment with DB, MA, and EP induced greater procollagen type I production than UVB-irradiated cells (Figure 1(a)). In addition, the cells treated with DB, MA, and EP had lower MMP-1 production, respectively, compared to UVB-irradiated fibroblasts (Figure 1(b)). These results provide evidence that the eight yin-tonic herbal medicines prevent the procollagen type I reduction and MMP-1 elevation which are observed after UVB irradiation. This result suggests that yin-tonic herbal medicines restore collagen levels by inhibiting the production of MMP-1.

### 3.3. Effect on Cell Viability of Yin-Tonic Herbal Medicines and UVB Irradiation in Human Skin Equivalent

**Figure 2:**
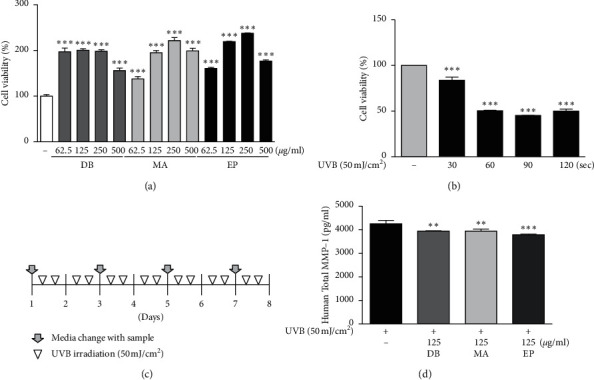
Effect on cell viability of yin-tonic herbal medicines and UVB irradiation in human skin equivalent model. (a) Cell viability of 3 yin-tonic herbal medicines and (b) UVB irradiation on the human skin equivalent. (c) The schematic diagram of UVB irradiation and treatment with yin-tonic herbal medicines using human skin equivalent model. (d) Effect of UVB irradiation time on MMP-1 production in human skin equivalent model. ^*∗∗*^*p* < 0.01 and ^*∗∗∗*^*p* < 0.001 versus nontreated group or UVB nonirradiated group.

To examine the effect of DB, MA, and EP on 3-dimensional skin, human skin equivalent was prepared. The MTT assay was used to determine the permissible concentration of DB, MA, and EP to be used on the human skin equivalent. The treatment with DB, MA, and EP at concentrations of 62.5, 125, 250, and 500 µg/ml increased cell viability, compared to nontreated cells (Figure 2(a)). To determine the optimal time condition of irradiation of UVB to the human skin equivalent, cell viability was evaluated after UVB irradiation (50 mJ/cm^2^) for 30, 60, 90, or 120 seconds, respectively, twice a day for 8 days. UVB irradiation decreased the cell viability, showing a rapid decline from UVB irradiation for 60 seconds in the human skin equivalent (Figure 2(b)). We also confirmed that UVB irradiation induced the decrease of procollagen and elevated MMP-1 production in condition of 30 to 60 seconds in the human skin equivalent (data not shown). It was determined that UVB irradiation (50 mJ/cm^2^) for 40 seconds considered the effect of UVB on the human skin equivalent and then conducted subsequent studies. The schematic diagram of UVB irradiation and treatment with yin-tonic herbal medicines using the human skin equivalent model is shown in Figure 2(c). The treatment with DB, MA, and EP significantly suppressed the UVB-induced MMP-1 production in UVB-irradiated human skin equivalent (Figure 2(d)).

### 3.4. Effect on Histological Changes in Skin of Yin-Tonic Herbal Medicine Extracts in UVB-Irradiated Human Skin Equivalent

**Figure 3:**
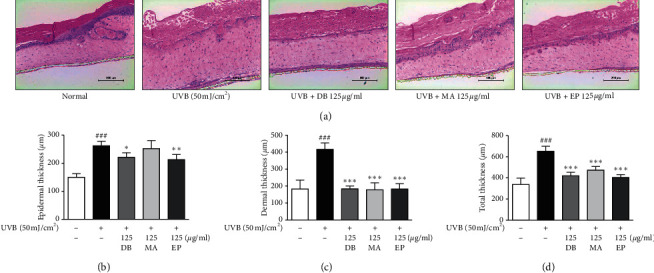
Effect on skin histological changes of yin-tonic herbal medicines in UVB-irradiated human skin equivalent model. (a) Histological features of the human skin equivalent. Samples were excised, fixed in 10% formaldehyde, embedded in paraffin, and sectioned. The sections were stained with H&E (scale bar = 200 *μ*m). (b) Epidermal, (c) dermal, and (d) total thickness in H&E stained sections were measured under a microscope. The data shown represent mean ± SD of three independent experiments. ^###^*p* < 0.001 versus the control group; ^*∗*^*p* < 0.05, ^*∗∗*^*p* < 0.01, and ^*∗∗∗*^*p* < 0.001 versus UVB-irradiated group.

**Figure 4:**
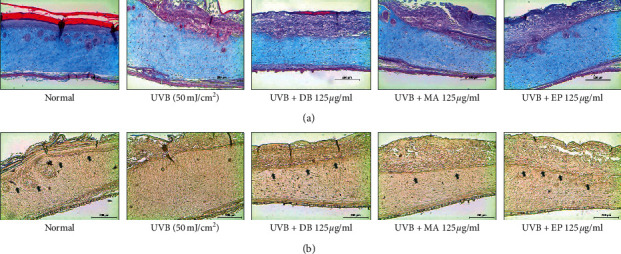
Effect on collagen and filaggrin expressions of yin-tonic herbal medicines in UVB-irradiated human skin equivalent model. (a) Masson's trichrome staining for the visualization of collagen fibers. Collagen fibers appear blue. (b) Skin sections were stained with immunohistochemical staining. Filaggrin in the skin equivalent was detected using specific antibodies. Black arrows indicated stained. Bars indicate 200 *μ*m.

Microscopically, epidermal thickening is another feature of photoaged skin. The effects of DB, MA, and EP on UVB-irradiated human equivalent skin were investigated histochemically. We observed the epidermis, dermis, total layer thickness, and collagen content. As expected, the thickness of skin equivalent tissue in the UVB-irradiated group was thicker than that of the unexposed group, but treatment with DB, MA, and EP decreased skin epidermal thickness in UVB-irradiated skin equivalent (Figure 3). UVB exposure resulted in severe epidermal necrosis, one of the features of sunburn, and abnormal desquamation which can lead to longer-term barrier disruption in UVB-irradiated human equivalent skin, while treatment with DB, MA, and EP mitigated this UVB-induced skin damage in UVB-irradiated skin equivalent (Figure 3). In addition, we observed intensity of collagen by Masson's trichrome staining. The collagen fibers of UVB-irradiated skin equivalent were less dense and more erratically arranged compared with the dense, regular fibers of nonirradiated skin. We also found that the accumulation of collagen in the dermis was prominent in the DB, MA, and EP-treated groups. In line with the *in vitro* results, collagen content was restored in the UVB-irradiated human skin equivalent (Figure 4(a)). These results suggest that DB, MA, and EP may block UVB-induced alterations of increased epidermal thickness and collagen degradation. The data showed that yin-tonic herbal medicines enhanced the function of collagen which is related to skin elasticity and moisture in the ECM of the dermis.

### 3.5. Effect on Filaggrin Expressions of Yin-Tonic Herbal Medicines in UVB-Irradiated Human Skin Equivalent

Filaggrin is a protein that is largely responsible for skin hydration and integrity of the skin barrier. This is essential for the production of natural moisturizing factor (NMF) substances that maintain skin hydration and skin barrier function [26]. In the skin aging process, several markers of epidermal differentiation including filaggrin were decreased, disturbing desquamation and the capacity of the stratum corneum to retain water [27]. The concentration of NMF components which are formed by filaggrin protein breakdown in the stratum corneum might be useful as a biomarker of the FLG genotype [28]. To study the effect of DB, MA, and EP on UVB-induced filaggrin protein expression, IHC was performed on human equivalent skin tissue sections. Filaggrin protein expression was attenuated by UVB irradiation on the near region of the epidermis of human equivalent skin tissue. UVB-irradiation samples treated with DB, MA, and EP exhibited intensified filaggrin protein levels compared with UVB-irradiated skin equivalent (Figure 4(b)). Upregulation of filaggrin might reduce skin aging and wrinkling, possibly by increasing moisture content and improving the skin barrier, indicating that yin-tonic herbal medicines can be expected to improve skin barrier function and moisturizing by increasing filaggrin in UVB-irradiated skin equivalent.

The root of *Dioscorea batata* Decaisne is neutral in nature, is sweet in flavor, and is utilized to cure yin deficiency in metabolic disorders, such as diabetes and hyperthyroidism, by tonifying and replenishing organs [29]. The fruit of *Morus alba* L. is commonly used as a tonic, sedative, and laxative herbal medicine [30]. *Eclipta prostrata* L. is also added to medicinal tonics that are usually used for treating dizziness, hemoptysis, hematuria, tinnitus, and uterine bleeding [31]. Considering those sedative, relieving, and moderate cooling properties, it is expected to be effective on photoaged skin. This is the first study to investigate the antiphotoaging effects of yin-tonic herbal medicines using human skin equivalent. The present study is important in furthering understanding of the role of yin-tonic herbal medicines on skin and photoaging in terms of identifying the scientific basis of herbal medicine background knowledge. However, there are some limitation in this study. First, we only considered fibroblast not keratinocytes *in vitro* study. Human keratinocytes are located in the outermost skin layer and therefore continuously exposed to UVR. It has been reported that the deleterious effects of UVB radiation on keratinocytes induce inflammation, cell death, senescence, and epithelial tumors [32]. Considering that the skin is a complex organ containing an intricate network, the antiphotoaging properties of yin-tonic herbal medicines on keratinocytes should be determined and discussed to integrate the antiphotoaging effect on skin. Second, this study is limited by the lack of information on the natural compounds that exert photoprotective effect. Plant extracts, natural compounds, or metabolites such as melatonin [33] and vitamin D compounds [34] have been considered as one of the most promising sources to prevent or ameliorate the effects of UVB. Some yin-tonic herbal medicines possess antioxidant properties, having rich antioxidants such as phenolic compounds, which contribute to the yin-tonic or antioxidative activity [35, 36] as well as anti-inflammatory properties [35], implying that yin-yang theory is somewhat equivalent to the modern theory of antioxidant-oxidant balance. It is known that some n irradiation on the skin suggests the large potential for growth of this field of study [37]. In addition, the primary mechanism by which UV irradiation initiates molecular responses in human skin is by photochronical generation of reactive oxygen species (ROS) [38]. Since antioxidants are capable of scavenging ROS, they behave as antiaging compounds in skin [39]. Recently, it has been suggested that natural products have shown antiphotoaging effects through its antioxidant and anti-inflammatory properties [40, 41]. Indeed, the antioxidant and anti-inflammatory effects of *D. batata* [42–44], *M. alba* [45, 46], and *E. prostrata* [47, 48] have been reported, which prevent prolonged skin damage and wrinkle formation. It is important to note that active compounds such as allantoin from *D. batata*, anthocyanin from *M. alba*, and wedelolactone from *E. prostrata*, exert the antioxidant and anti-inflammatory effects [49–51], supporting the photoprotective effect of yin-tonic herbal medicines in this study. Given this fact, further studies are needed to identify the molecular mechanisms involved in ROS level and DNA protection against oxidant challenge, moreover, to better understand the protective roles of yin-tonic herbal medicines and derived compounds from the deleterious effects of UVB radiation.

## 4. Conclusions

In conclusion, the yin-tonic herbal medicines provided potential antiphotoaging and antiwrinkle activities *in vitro* and showed a significant antiphotoaging effect including the restoration of collagen and filaggrin expression on human skin equivalent *in vivo*. Overall, the results obtained in this study suggested the promising complementary materials for improving skin and contributed to elucidating the mechanisms of antiphotoaging properties of yin-tonic herbal medicines. We believe that our findings could be used to help better understand the antiaging effect of yin-tonic herbal medicine and identify the scientific basis of herbal medicine background knowledge.

## Figures and Tables

**Table 1:** List of eight yin-tonic herbal medicines.

No	Scientific name	Remarks	Yield (%)
1	Extract of *Adenophora triphylla* var. japonica Hara	AT	33.22
2	Extract of *Liriope platyphylla* Wang et Tang	LP	39.88
3	Extract of *Rehmannia glutinosa* liboschitz var. purpurea Makino	RG	13.81
4	Extract of *Scrophularia buergeriana* Miquel	SB	23.16
5	Extract of *Dioscorea batata* Decaisne	DB	26.72
6	Extract of *Morus alba* Linne	MA	7.13
7	Extract of *Eclipta prostrata* Linne	EP	9.73
8	Extract of *Cistanche deserticola* Y. C. Ma	CD	30.96

**Table 2:** Effect on cell viability (%) of eight yin-tonic herbal medicines on HaCaT keratinocytes.

Concentration (*μ*g/ml)	Cell viability (%)			
	AT	LP	RG	SB
0	100.00 ± 2.38	100.00 ± 2.38	100 ± 2.38	100 ± 2.38
7.8	110.51 ± 3.49^*∗*^	111.86 ± 2.69^*∗∗*^	113.95 ± 2.91^*∗∗∗*^	117.61 ± 3.34^*∗∗∗*^
15.6	112.16 ± 3.02^*∗∗*^	116.22 ± 6.28^*∗∗∗*^	120.92 ± 0.42^*∗∗∗*^	119.01 ± 1.98^*∗∗∗*^
31.3	110.29 ± 3.91^*∗*^	118.53 ± 2.30^*∗∗∗*^	117.83 ± 3.86^*∗∗∗*^	118.48 ± 0.91^*∗∗∗*^
62.5	114.08 ± 7.85^*∗∗*^	118.53 ± 5.14^*∗∗∗*^	122.84 ± 2.45^*∗∗∗*^	128.20 ± 4.06^*∗∗∗*^
125	115.48 ± 0.94^*∗∗*^	115.95 ± 3.93^*∗∗∗*^	121.80 ± 1.87^*∗∗∗*^	133.52 ± 2.06^*∗∗∗*^
250	117.44 ± 4.50^*∗∗∗*^	119.22 ± 2.60^*∗∗∗*^	119.18 ± 4.26^*∗∗∗*^	128.77 ± 3.21^*∗∗∗*^
500	139.71 ± 0.79^*∗∗∗*^	128.03 ± 2.80^*∗∗∗*^	129.90 ± 5.07^*∗∗∗*^	118.26 ± 4.52^*∗∗∗*^

Concentration (*μ*g/ml)	Cell viability (%)			
	DB	MA	EP	CD
0	100.00 ± 1.23	100.00 ± 1.23	100 ± 1.23	100 ± 1.23
7.8	128.90 ± 4.94^*∗∗∗*^	138.88 ± 4.62^*∗∗∗*^	139.61 ± 7.28^*∗∗∗*^	113.01 ± 9.72
15.6	138.98 ± 2.70^*∗∗∗*^	158.46 ± 0.60^*∗∗∗*^	166.22 ± 5.57^*∗∗∗*^	159.51 ± 5.72^*∗∗∗*^
31.3	142.46 ± 4.20^*∗∗∗*^	167.64 ± 2.57^*∗∗∗*^	176.46 ± 5.20^*∗∗∗*^	155.24 ± 14.0^*∗∗∗*^
62.5	134.55 ± 8.47^*∗∗∗*^	172.39 ± 8.60^*∗∗∗*^	182.21 ± 7.40^*∗∗∗*^	152.44 ± 5.31^*∗∗∗*^
125	145.63 ± 2.40^*∗∗∗*^	187.54 ± 10.70^*∗∗∗*^	199.21 ± 7.66^*∗∗∗*^	163.90 ± 3.16^*∗∗∗*^
250	156.82 ± 7.48^*∗∗∗*^	192.50 ± 6.55^*∗∗∗*^	229.03 ± 3.11^*∗∗∗*^	173.24 ± 1.61^*∗∗∗*^
500	182.74 ± 4.69^*∗∗∗*^	190.18 ± 4.02^*∗∗∗*^	265.24 ± 7.85^*∗∗∗*^	171.76 ± 5.36^*∗∗∗*^

The data shown represent mean ± S.D. of three independent experiments. ^*∗*^*p* < 0.05,^*∗∗*^*p* <  0.01,^*∗∗∗*^*p* < 0.001 vs. untreated group.

**Table 3:** Effect on cell viability (%) of eight yin-tonic herbal medicines on CCD-986sk fibroblast.

Concentration (*μ*g/ml)	Cell viability (%)			
	AT	LP	RG	SB
0	100.00 ± 1.02	100.00 ± 1.02	100 ± 1.02	100 ± 1.02
7.8	92.97 ± 2.26^*∗∗∗*^	92.67 ± 0.47^*∗∗∗*^	91.51 ± 0.69^*∗∗∗*^	97.44 ± 1.54
15.6	92.65 ± 1.33^*∗∗∗*^	89.29 ± 0.87^*∗∗∗*^	91.66 ± 0.22^*∗∗∗*^	95.19 ± 3.24
31.3	93.08 ± 1.33^*∗∗∗*^	91.37 ± 1.60^*∗∗∗*^	91.80 ± 1.96^*∗∗∗*^	94.12 ± 3.27^∗^
62.5	98.16 ± 3.18	93.25 ± 1.10^*∗∗∗*^	91.32 ± 2.03^*∗∗∗*^	93.30 ± 0.00^*∗*^
125	93.88 ± 0.21^*∗∗*^	93.11 ± 0.47^*∗∗∗*^	91.95 ± 2.47^*∗∗∗*^	95.77 ± 1.89
250	94.68 ± 0.10^*∗∗*^	96.06 ± 1.40^*∗∗*^	96.74 ± 1.30	99.11 ± 2.87
500	88.73 ± 1.74^*∗∗∗*^	89.67 ± 0.62^*∗∗∗*^	100.99 ± 2.87	104.14 ± 3.06

Concentration (*μ*g/ml)	Cell viability (%)			
	DB	MA	EP	CD
0	100.00 ± 1.99	100.00 ± 1.99	100 ± 1.99	100 ± 1.99
7.8	87.49 ± 2.52^*∗∗∗*^	87.94 ± 1.95^*∗∗∗*^	87.27 ± 1.62^*∗∗∗*^	96.19 ± 2.52
15.6	91.31 ± 2.99^*∗∗∗*^	87.13 ± 0.94^*∗∗∗*^	90.12 ± 0.61^*∗∗∗*^	95.97 ± 2.82
31.3	91.62 ± 0.30^*∗∗∗*^	87.70 ± 1.92^*∗∗∗*^	88.03 ± 1.23^*∗∗∗*^	90.77 ± 1.11^*∗∗∗*^
62.5	90.40 ± 2.22^*∗∗∗*^	89.27 ± 1.03^*∗∗∗*^	87.99 ± 1.54^*∗∗∗*^	97.04 ± 2.72
125	92.62 ± 0.10^*∗∗*^	90.51 ± 1.46^*∗∗∗*^	92.03 ± 1.76^*∗∗∗*^	98.97 ± 1.21
250	91.84 ± 2.42^*∗∗∗*^	93.31 ± 1.44^*∗∗∗*^	99.54 ± 1.40	103.39 ± 2.02
500	101.44 ± 0.58	99.87 ± 1.20	114.30 ± 0.91^*∗∗∗*^	117.72 ± 0.71^*∗∗∗*^

The data shown represent mean ± S.D. of three independent experiments. ^*∗∗*^*p* <  0.01 and ^*∗∗∗*^*p* < 0.001 versus untreated group.

## Data Availability

The data used to support the findings of this study are available from the corresponding author upon request.
